# Positron Annihilation Spectroscopy Study of Metallic Materials after High-Speed Cutting

**DOI:** 10.3390/ma15031017

**Published:** 2022-01-28

**Authors:** Jinquan Li, Roman Laptev, Iurii Bordulev, Krzysztof Siemek, Pawel Horodek, Haolun Shen, Anton Lomygin, Jian Cui

**Affiliations:** 1 School of Mechanical Engineering, Shenyang Ligong University, No.6 Nanping Center Road, Hunnan New District, Shenyang 110159, China; shenhaolun0127@163.com; 2 Division for Experimental Physics, National Research Tomsk Polytechnic University, 634050 Tomsk, Russia; bus@tpu.ru (I.B.); lomyginanton141@gmail.com (A.L.); ttszyan@mail.ru (J.C.); 3 Institute of Nuclear Physics Polish Academy of Sciences, PL-31342 Krakow, Poland; siemek.krzysztof@gmail.com (K.S.); pawel.horodek@ifj.edu.pl (P.H.); 4 Joint Institute for Nuclear Research, Joliot-Curie 6, 141980 Dubna, Russia

**Keywords:** positron annihilation, metals, high-speed cutting, white layer, microstructure, defects

## Abstract

During high-speed cutting, a white layer is often produced on the machined surfaces after mechanical machining, seriously affecting the mechanical properties. These properties are related to the material structure and the defects induced by cutting. However, there is a lack of research on the atomic-scale defects of the white layer. This paper studied the influence of cutting parameters, namely the feed rate, cutting speed and cutting depth, on atomic-scale defects induced by high-speed cutting in GCr15 steel. Positron annihilation studies showed typical plastically deformed or tempered carbon steel defects with additional vacancy cluster components. The quantity of these clusters changed with cutting parameters. Furthermore, significant changes were observed in the subsurface region up to 1 µm, occurring as a result of simultaneous phase transformations, deformation and thermal impacts. The predominant accumulation of only one type of atomic-scale defect was not observed.

## 1. Introduction

During the high-speed cutting process, a tool and a machined surface instantly collide at high speed, causing the temperature of the partial area to rise sharply. Under the action of the cutting edge of the tool, the surface layer of the machined material is subjected to the solid thermo-mechanical coupling effect, which gives rise to the deformed thermal layer on the machined surface. This effect has a significant influence on the mechanical properties of the workpiece [1]. The plastic deformation is most significant on the surface of the deformed thermal layer, and gradually decreases below the machined surface until a certain depth at which its microstructure is consistent with the matrix material. The microstructures and mechanical properties of the degenerating layer vary with different processing methods. During the high-speed cutting process, for many metal materials, a layer that appears white under an optical microscope is produced on top of the degenerating layer, caused by the machining of the workpiece’s surface [2]. This ultrafine-grain white layer has a surface hardness much higher than that of the matrix, ranging from 800 to 1200 HV, while its thickness ranges from 10 to 50 μm [3]. The high brittleness of the white layer may allow cracks to form, making it possible for the white layer to fall off and become a fatigue source, dramatically reducing the fatigue limit of the structure piece [4].

Thus far, the formation mechanism of the white layer has been studied in depth. Chou and Evans [5] suggested that during the machining process of iron-based alloys, the temperature of the machined surface rises sharply due to the intense friction between the tool and the chip, reaching the A_C__1_ transformation temperature to produce austenitization. The heat then decreases sharply as the chip peels, rapidly chilling and quenching the surface of the workpiece, and produces a phase change as austenite is transformed into fine-grained martensite. The white layer is simply a high-density structure composed of these sub-grains and recrystallized grains. However, research indicates that the white layer can also be formed below the phase transition temperature as a result of strong plastic deformation during the cutting process. The plastic deformation results in grain refinement, which promotes the formation of the white layer [6]. The study of Bulpett et al. [7] showed that the white layer is formed by the combined effects of the phase transformation at a high temperature and the plastic deformation. The heat generated during the processing raises the partial temperature to the phase transition temperature, while the grain can simultaneously be refined by the strong plastic deformation. Barry and Byrne [8] reported that during the high-speed cutting process, under the action of significant strain and a high strain rate, severe plastic deformation in the contact area between the tool and the machined surface produces many dislocations. The dislocation density within the material thus increases sharply. The plastic work accumulates gradually, and the heat is released slowly. The rapid temperature rise is exceptionally high. When the processed surface reaches the dynamic recrystallization temperature, the deformed grains grow into new grains around a core of sub-crystalline material with a higher dislocation density or impurity phases to form the white layer.

Existing research into the formation mechanism of the white layer during the high-speed hard cutting process is primarily concentrated on two aspects [9,10]: the phase transformation caused by the thermal load and the plastic deformation caused by the mechanical load. There are four main viewpoints [3,8,11], as follows. (1) Mechanism of thermal action: it is believed that the formation of the white layer results from the metal phase transition caused by the mechanism of thermal action. (2) Plastic deformation mechanism: it is believed that the formation of the white layer is unrelated to the thermal effect. Instead, strong plastic deformation leads to grain refinement and thus promotes the formation of the white layer. (3) Dynamic recrystallization mechanism: when the machined surface reaches the dynamic recrystallization temperature, new grains are produced to form the white layer. (4) The coupling mechanism of thermal–plastic deformation: the high temperature in the partial area of the machined surface reaches or exceeds the phase transition temperature, and the strong plastic deformation of the machined surface causes the grain refinement to generate high pressure, which results in a reduction in the critical phase transition temperature to promote the generation of the white layer. The white layer thus results from the coupling effect of temperature and plastic deformation.

Summarizing the current research on the formation mechanism of the surface layer, the microstructures of the white layer observed by electronic microscopes are usually martensite, austenite, ultra-fine grains, high-density dislocations, phase boundaries, grain boundaries, twinning, slip line, and so on, which form a relatively macroscopic set of organization, defects, and various phase transformation structures. Experimentally, conventional testing methods are typically used, such as optical microscopy, scanning electron microscopy (SEM), transmission electron microscopy (TEM), X-ray diffraction, scanning tunnelling electron microscopy, and backscattered electron imaging. The most frequently used methods are SEM and TEM. However, SEM is only used to observe surface topography, and cannot detect subsurface or interface defects. TEM is also used to indirectly examine dislocation and other defects based on the electron diffraction pattern, and it is difficult to produce samples featuring dislocation. In terms of theoretical analysis and numerical simulation, the study of the microstructures of the white layer is based on continuum mechanics. Although valuable results have been obtained, it is difficult to characterize partial defect information at the atomic scale using these methods [12], and it is impossible to study interface characteristics and the relationship between microstructural defects and macroscopic properties at the atomic level—this requires further research at the electronic structure level to understand the nature of the problem. The design and choice of the alloy material from the atomic or electronic scale is one of the most active frontiers of current material science. At present, positron annihilation technology is an indispensable method for studying material defects and phase transitions due to its sensitivity to the atomic-scale defects in samples and its non-destructive detection characteristics, and it has been widely used in many fields, such as solid-state physics and materials science [13]. Therefore, positron annihilation technology may compensate for the lack of current research methods regarding the formation mechanism of the white layer.

The types and concentrations of defects can be measured using positron annihilation, which can be used to study the distribution of elements around or within defects, as well as the electronic structure characteristics. It can also be used to study the characteristics of defect configurations and the interaction mechanism among the added elements, impurity atoms, and micro-defects [14]. In particular, positron annihilation technology plays an irreplaceable role in analyzing the mechanisms of crystallization and precipitation caused by the migration of trace elements with the defects in the material.

Information on the electron momentum distribution in a portion of the impurity, the precipitation deposition phase, and the elemental momentum distribution can be obtained by using the effect that the positrons are trapped by impurity clusters. The diffusion, precipitation, deposition of impurities in the material, and cluster growth mechanism significantly impact the mechanical properties of metals and metal alloys. All these microstructure characteristics are difficult to observe via high-resolution electron microscopy, so positron annihilation spectroscopy (PAS) offers a promising new experimental analysis method.

Consequently, positron annihilation technology has been widely used in many study fields to explore a range of effects, including the defects on the atomic scale, such as vacancies, caused by object deformation and quenching; the deformation, phase transition, hydrogen embrittlement [15,16], and fatigue damage in the initial stage for metals and alloys; the defect types and scales located in the surface and interface structures [17]; the microstructure characterization of various functional materials [18]; and the mechanisms of the formation and evolution of vacancies in semiconductor materials [19,20,21].

In this paper, the characteristics of the atomic-scale defects in the white layer and bulk of GCr15 steel were studied using positron annihilation spectroscopy techniques to make the research on the formation mechanism of the white layer more complete.

## 2. Materials and Methods

### 2.1. Cutting Test

The single-factor dry cutting experiment was conducted on a MULTUS B400-W CNC lathe. PCBN cubic boron nitride blades were used. The tool rake angle was *γ*_0_ = 5°, the rear angle was *α*_0_ = 7°, the cutting edge angle and inclination were *k_r_* = 90° and *λ_s_* = 0°, respectively, and the corner radius was *r_c_* = 0.8 mm. The test material was GCr15 bearing steel that was heated to 850 °C, held for 2 h, quenched with 10% saltwater, tempered at 320 °C, and then held for 4 h, with a hardness of HRC60. Its chemical composition is shown in Table 1.

**Figure 1:**
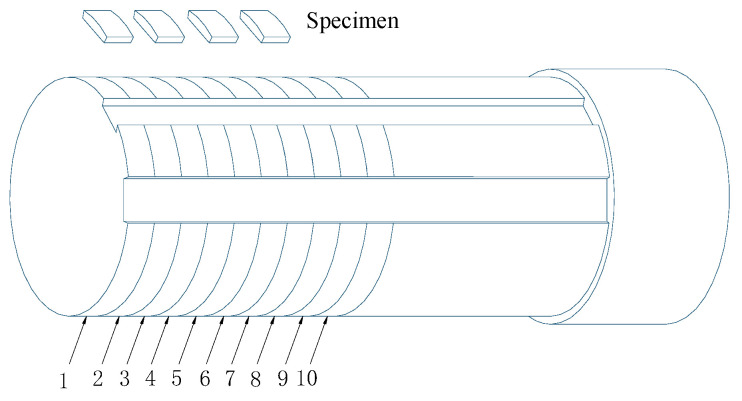
A schematic diagram of the surface layer sample.

In total, the workpiece was 300 mm in length and 90 mm in diameter. The specimen was divided into several segments (Figure 1) with the corresponding cutting parameters (see the numbered list in Table 2) used along the length of the workpiece. The surface layer of each segment on the sample was cut into several portions, as shown in Figure 1. The sample size was 10 × 10 × 2 mm.

### 2.2. Positron Annihilation Experiment

PAS is a promising method for analyzing the defect structure of materials during high-speed cutting, allowing researchers to study the mechanisms and control the dynamics of the appearance, transformation, and disappearance of defects of different dimensions (from point to extended) in a wide range of concentrations. Positron lifetime studies allow the type, size, and concentration of defects to be determined, and, when combined with Doppler annihilation line broadening spectroscopy (DBS), allow the observation of phase transformations and analysis of the chemical environment at the annihilation site. The mean positron implantation depth emitted from the ^44^Ti isotope into steel is around 150 µm. To analyze the surface and the subsurface region (up to ~1.2 μm), it is necessary to use a variable-energy positron beam. The physical bases and features of the applications of PAS are presented in detail in a number of modern reviews [22,23,24].

The white layer was studied using the Doppler broadening of the annihilation line using the variable positron energy at the JINR DLNP in Dubna, Russia [25,26]. A monoenergetic flux of positrons with a diameter of 5 mm and an intensity of 10^6^ e^+^/s was used. The energy range of the implanted positrons ranged from 0.1 to 36 keV. The Doppler broadening measurements were performed and annihilation γ-radiation was detected by an HPGe detector model GEM25P4-70 (AMETEK ORTEC, USA) with an energy resolution of 1.20 keV, interpolated for the energy of 511 keV. The obtained DBS spectra were analyzed by extracting the S and W parameters, defined as the zone below the central or wing parts of the annihilation line divided by the total area below this line, respectively.

For bulk analysis, complex spectroscopy based on the positron annihilation lifetime spectrometry (PALS) module and Coincidences Doppler broadening spectroscopy (CDBS) were used [27,28]. The temporal resolution of the PALS module was 230 ± 6 ps, with a count rate of 105 ± 30 events/s. The count rate for the CDBS module was 130 ± 20 events/s at an energy resolution of 1.15 ± 0.04 keV. The radioactive isotope ^44^Ti was used as a positron source, with an activity of 1.38 MBq and maximum energy of 1.47 MeV. For each sample, two PALS spectra and one two-dimensional CDBS spectrum were collected, with the statistics of 3 × 10^6^ and 18 × 10^6^ events, respectively. The PALS spectra were analyzed by the ‘four-state trapping model’ using the LT10 software (version 10.2.2.2) [29,30,31]. In this model, the spectrum is analyzed using four time components, τ_A_, τ_B_, τ_C_, and τ_F_; intensities, I_A_, I_B_, and I_C_; trapping rates, k_A_, k_B_, and k_C_; and the average positron lifetime τ_avg_. The first three components correspond to the positron annihilation in A, B, and C states, while τ_F_ corresponds to the lifetime of a delocalized positron in the lattice GCr15. The lifetime components were partially common for all samples. The contribution of the positron source was determined using an empirical function and amounted to approximately 9%. The time components of positron annihilation in the source were equal: τ_1_ = 142 ps (48.2%), τ_2_ = 468 ± 3 ps (42.8%), and τ_3_ = 2800 ± 27 ps (9.0%).

The obtained DBS spectra were analyzed by extracting the S and W parameters. These parameters were obtained by processing the spectra in the CDBTools software [32]. To analyze the two-dimensional spectrum, relative curves were plotted as R(E) = N(E)/N_0_(E), where N(E) is the spectrum of the sample under study and N_0_(E) is the spectrum of the reference sample. A defect-free Fe sample was used as a reference material for N_0_(E). The spectra of annealed samples of silicon, chromium, carbon, copper, and nickel were also used for constructing the relative curves.

## 3. Results and Discussion

PALS and DBS spectra for GCr15 steel samples after high-speed cutting with different parameters, and two-dimensional CDBS spectra, are given in the Supplementary Materials.

### 3.1. Influence of the Cutting Speed

The PALS experimental results for GCr15 samples as a function of the cutting speed are shown in Table 3.

All samples’ spectra contain four time components: τ_F_ = 106 ± 6 ps, τ_A_ = 147 ± 1 ps, τ_B_ = 240 ± 4 ps, and τ_C_ = 1.75 ± 0.04 ns (τ_C_ is associated with the annihilation of positrons in the sample holder and was not used in further analysis; I_C_ < 1.2%). The time component τ_F_ with a lifetime of 106 ± 6 ps corresponds to the theoretical and experimental values of the lifetime of delocalized positrons in the iron crystal lattice [33]. The component τ_A_ = 147 ± 2 ps is very close to the experimental values of positrons trapped by plastic deformation-induced defects [34]. These can be dislocations or defects associated with them, such as jogs, vacancies [35,36,37], and vacancy–solute complexes for steel components (e.g., v-Cr (153 ps), v-Mn (152 ps), v-Cu (153 ps), and v-C (160 ps)) [38,39,40]. Moreover, similar positron lifetimes were found for martensite (159 ± 3 ps), bainite (164 ± 3 ps), pearlite (151 ± 3 ps), and austenite (149 ± 3 ps) steel structures [41,42] after different heat treatments. This implies that this lifetime cannot be unequivocally assigned to only one type of defect.

Unambiguous identification of the second time component, τ_B_ = 240 ± 4 ps, is also problematic. A similar value is typical for vacancy clusters (~5) in the Fe lattice [35,36,37,42,43], as well as defects associated with second-phase precipitates (e.g., the Fe_3_C cementite–matrix interface [44]). Comparable values are also found for iron and chromium oxides, the formation of which is often due to natural oxidation (rust) and annealing in the air [45]. It is known that the presence of large clusters of defects is related to the cracking mechanism and may adversely affect the mechanical properties of the white layer. Analysis of PALS spectra alone does not allow the determination of the nature of positron trapping centers in steel after high-speed cutting; therefore, it is necessary to supplement it with DBS and CDBS data.

**Figure 2:**
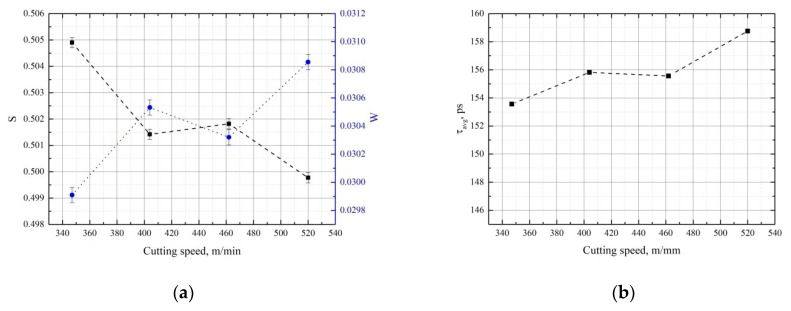
Dependencies of S (black) and W (blue) parameters (**a**) and average positron lifetime (**b**) as a function of cutting speed for GCr15 steel.

Figure 2 shows the DBS parameters (a) and the average positron lifetime (b) for GCr15 steel samples as a function of cutting speed.

It is clearly seen that the S parameter and the mean lifetime have opposite trends, which is unusual, as both parameters are sensitive to the free volume defects in similar ways [46,47]. This behavior indicates the significant influence of the chemical environment (e.g., secondary phase precipitate formation) or phase transformations (e.g., transformation of martensite to austenite) [42]. Increasing the cutting speed of GCr15 steel produces intense heating above the austenitic transformation temperature and promotes secondary quenching in which cryptocrystalline martensite and cementite are formed, thus causing the S parameter to decrease and the average lifetime of the positrons to increase.

**Figure 3:**
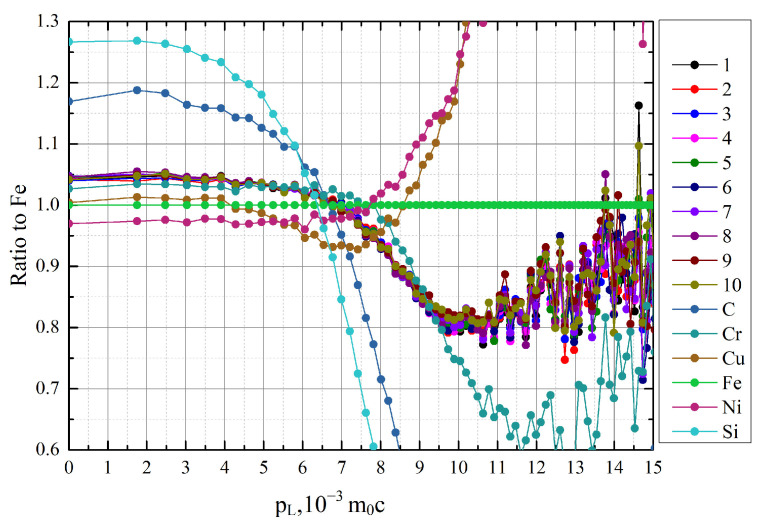
Ratio curves to Fe for GCr15 samples with different cutting parameters and steel components. Description of samples is given in Table 1.

The CDB ratio curves for pure annealed iron obtained from all the studied samples of GCr15 steel are shown in Figure 3, along with the ratio curves for the major alloying elements (C, Si, Cu, Ni, and Cr).

The shift in the CDB ratio lines towards the CDB signature curves of pure alloying elements can be interpreted as an association of the defects with these alloying elements. The manganese line is not shown in the figure, but since the ground-state electronic configuration of Mn (4s^2^ 3d^5^) is almost the same as that of Cr (4s^1^ 3d^5^), and Cr possesses similar positron affinity to Mn, their ratio curves will nearly coincide across the range [41]. All GCr15 steel samples, regardless of the cutting parameters, are characterized by a shifting towards carbon in both the low- and high-momentum regions. These results could indicate that larger amounts of carbon (with a contribution from Mn or Cr) are bound to vacancy-type defects. This agrees well with the PALS data and could be caused by the existence of several types of structures related to the traps, rather than a high concentration of a single type of defect.

**Figure 4:**
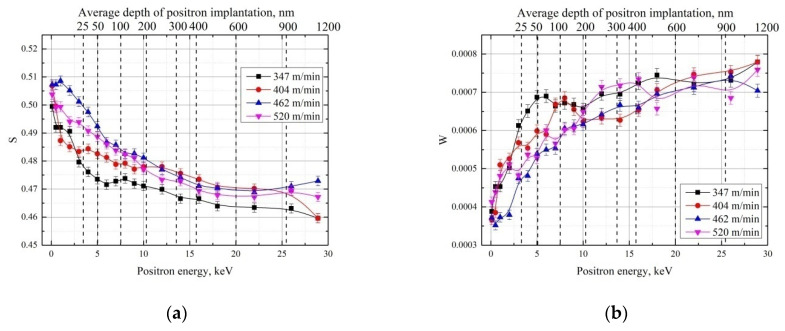
DBS-VEP depth profile of the S (**a**) and W (**b**) parameters for the white layer in GCr15 steel samples with different cutting speeds.

The results of layer-by-layer analysis using variable-energy positron beams are shown in Figure 4. The characteristic increase in the S parameter for low-energy positrons observed in all spectra is caused by the increased number of positrons annihilated from the surface state. Above 15 keV in energy, all positrons are annihilated inside the material, and only a negligible number of positrons can diffuse and annihilate on surface defects. Due to the difficulty of assigning changes to only one type of defect, these changes will only be interpreted in terms of the difference between cutting parameters. Typically, the S parameter can be considered an integral characteristic representing the total open volume defect level, but, as shown above, in the case of high-speed steel cutting, it is also influenced by phase transformations. The phase transformation from the martensitic to austenitic phase is accompanied by a decrease in the S parameter [41,48]. The reverse martensitic transformation leads to the S parameter increasing. Thus, it is not possible to separate the contribution from phase transformations in steel during high-speed cutting that are associated with the accumulation of deformation or quench defects, and hereinafter we will consider only their cumulative effect.

The GCr15 steel samples cut at the minimum cutting speed (347 m/min) are characterized by the minimum S parameter and maximum W up to 1.2 μm depth. An increase in cutting speed by 16.5% leads to a rise in the S parameter, accompanied by a decrease in the W term at depths from 25 to 900 nm. Increasing the cutting speed to 462 m/min then leads to a further increase in the S parameter, with the most noticeable differences occurring at depths up to 30 nm. At the highest cutting speed of 520 m/min, there is a decrease in the S parameter compared to the speed of 462 m/min, but only at depths up to 50 nm. At greater depths, there are no noticeable changes. The results agree with the data presented in [49], which demonstrate that with the increase in cutting speed, the retained austenite content tends to first increase and then decrease.

### 3.2. Influence of the Feed Rate

The PALS experimental results for GCr15 samples, showing the dependence on the feed rate, are shown in Table 4.

The PALS data at different feed rates identify the same lifetime components as at different cutting speeds (see Table 3). Increasing the feed rate first increases the intensity of the component τ_A_, and then decreases it below the starting level. The intensity variation of the second component τ_B_ has no clear trend.

**Figure 5:**
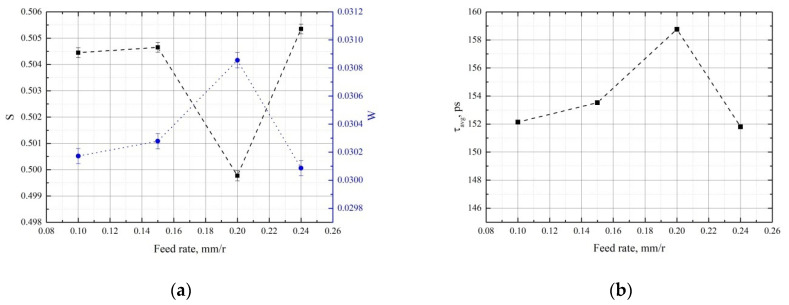
Dependencies of S (black) and W (blue) parameters (**a**) and average positron lifetime (**b**) as a function of feed rate for GCr15 steel.

Figure 5 presents the DBS parameters (a) and the average positron lifetime (b) for GCr15 steel samples as a function of feed rate.

The graphs of the S parameter and the average lifetime as a function of the feed rate are also reversed. In this case, the average positron lifetime increases, first insignificantly as the feed rate is changed from 0.1 to 0.15 mm/r, and then dramatically at 0.2 mm/r. It then decreases significantly at the maximum feed rate. The chemical environment at the positron annihilation site does not change (see Figure 3). Thus, it can be assumed that an increase in the feed rate from 0.1 to 0.2 mm/r is accompanied by an increase in the content of the martensitic phase in the bulk of GCr15 steel, while, at 0.24 mm/r, the austenitic phase is already growing.

**Figure 6:**
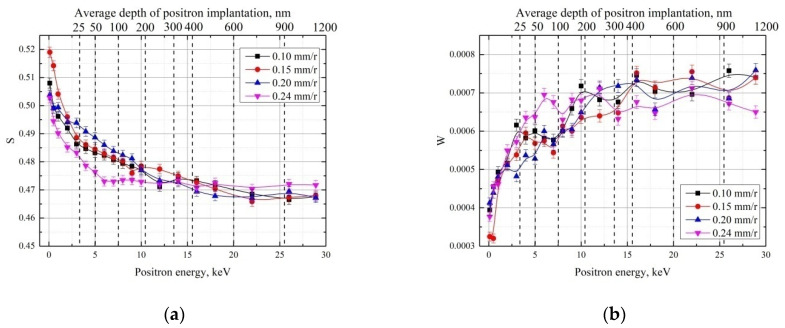
DBS-VEP depth profile of the S (**a**) and W (**b**) parameters for the white layer in GCr15 samples with different feed rates.

The DBS results of the white layer analysis using variable-energy positron beams are shown in Figure 6.

Increasing the feed rate from 0.1 to 0.2 mm/r does not lead to observable changes in the S and W parameters throughout the range of implanted positron energies. However, at ***f*** = 0.24 mm/r, a decrease in the S parameter at depths up to 200 nm is observed. This may indicate an increase in martensitic phase content in this region.

### 3.3. Influence of the Cutting Depth

The dependence of the PALS experimental results on the cutting depth for GCr15 samples is shown in Table 5.

Variation in the cutting depth affects the parameters of the positron annihilation time characteristics differently. As the depth increases up to 0.15 mm, I_A_ decreases, while I_B_ increases. Between 0.15 and 0.2 mm, the intensities of both components rise. However, at 0.25 mm, this trend persists only for the first component, while I_B_ decreases. These changes also indicate that the variation in high-speed cutting parameters does not simply induce changes in one type of defect, but rather gives rise to a cascade of complex microstructural transformations, which have different effects on the defect structure and positron trapping centers.

**Figure 7:**
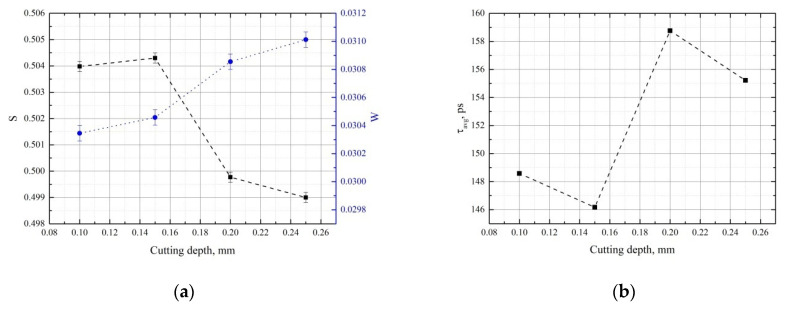
Trends in S (black) and W (blue) parameters (**a**) and average positron lifetime (**b**) as a function of cutting depth for GCr15 steel.

Figure 7 shows the DBS parameters (a) and the average positron lifetime (b) for GCr15 steel samples as a function of cutting depth.

Over cutting depths of 0.1 to 0.15 mm, there is no noticeable change. However, at 0.2 mm, S = *f*(*a_p_*) and τ_avg_ = *f*(*a_p_*) dependences show sharp reversals. This suggests that the plastic deformation occurring at this cutting depth has a strong influence on the phase transition (martensite to austenite). A further increase to 0.25 mm has no significant effect.

**Figure 8:**
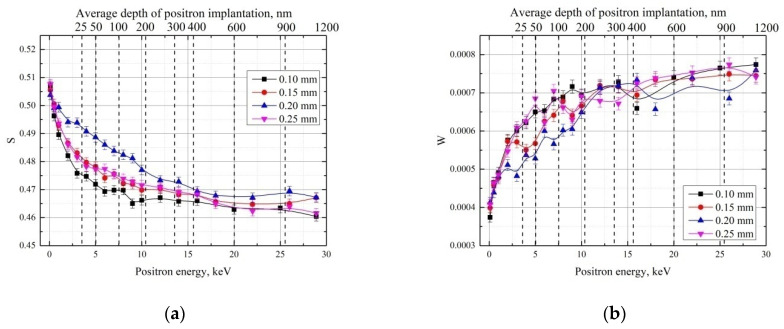
DBS-VEP depth profile of the S (**a**) and W (**b**) parameters for the white layer in GCr15 steel samples with different cutting depths.

The positron beam analysis of the white layer depending on cutting depth is shown in Figure 8.

Changing the cutting depth has a notable effect on the subsurface zone of the white layer in GCr15. For all energies, the minimum cutting depth is characterized by the lowest S parameter value of the implanted positrons. Increasing the cutting depth to 0.15 mm raises this value at the positron implantation depth range from ~10 to 300 nm. As in the bulk analysis, sharp changes in the S parameter are observed at *a_p_* = 0.2 mm, increasing for the subsurface region and decreasing for the bulk. Increasing the cutting depth to 0.25 mm causes an inverse displacement for the S line, placing it at the same level as that of the 0.15 mm cutting depth.

## 4. Conclusions

The positron annihilation studies show that the white layer and bulk feature two major classes of defects. The first class is typical of tempered or plastically deformed steels, and its defects persist for a lifetime of around 147 ps. The second class is related to vacancy clusters. Larger clusters can negatively affect the white layer’s properties and can contribute to its brittleness, potentially causing it to fall off. A variation in the cutting parameters has a different effect on plastic deformation, and the phase transition is observed in the near-surface zone. Therefore, adequately chosen cutting parameters can limit the number of vacancy clusters. The lowest prevalence of these defects was observed from the studied parameters for a cutting depth of 0.1 mm, feed rate of 0.15 mm/r, and cutting speed of 347 m/min.

## Figures and Tables

**Table 1:** The chemical composition of GCr15 (wt %).

Material	C	Si	Mn	P, S	Cr	Ni	Mo
GCr15	0.95–1.05	0.15–0.35	0.25–0.45	0.025	0.40–1.65	Max 0.3	Max 0.10

**Table 2:** The cutting parameters.

Numbered List	Cutting Speed (*V*) (m/min)	Feed Rate (*f*) (mm/r)	Cutting Depth (*a_p_*) (mm)
1	520	0.20	0.25
2	520	0.20	0.20
3	462	0.20	0.20
4	404	0.20	0.20
5	347	0.20	0.20
6	520	0.10	0.20
7	520	0.15	0.20
8	520	0.24	0.20
9	520	0.20	0.15
10	520	0.20	0.10

**Table 3:** PALS parameters of GCr15 samples depending on cutting speed.

Cutting Speed, m/min	χ^2^	τ_A_, ±2 ps	τ_B_, ±4 ps	τ_F_, ±6 ps	k_A_, ns^−1^	k_B_, ns^−1^	I_A_, %	I_B_, %	τ_avg_, ps
347	1.16	147	240	106	19.1 ± 0.4	0.04 ± 0.08	86.7	0.2	149
404	1.09				15.4 ± 0.1	0.21 ± 0.03	83.4	1.0	146
462	1.12				21.0 ± 0.2	0.96 ± 0.04	84.0	3.5	159
520	1.17				22.2 ± 0.5	0.61 ± 0.05	84.9	2.3	155

**Table 4:** PALS parameters of GCr15 samples depending on feed rate.

Feed Rate, (mm/r)	χ^2^	τ_A_, ±1 ps	τ_B_, ±4 ps	τ_F_, ±6 ps	k_A_, ns^−1^	k_B_, ns^−1^	I_A_, %	I_B_,%	τ_avg_, ps
0.10	1.18	147	240	106	17.4 ± 0.1	0.47 ± 0.05	83.6	2.0	152
0.15	1.26				21.1 ± 0.1	0.39 ± 0.01	86.2	1.4	154
0.20	1.12				21.0 ± 0.2	0.96 ± 0.04	84.0	3.5	159
0.24	1.13				15.5 ± 0.2	0.55 ± 0.02	81.8	2.5	152

**Table 5:** Dependence of PALS parameters of GCr15 samples on cutting depth.

Cutting Depth, (mm)	χ^2^	τ_A_, ±1 ps	τ_B_, ±4 ps	τ_F_, ±6 ps	k_A_, ns^−1^	k_B_, ns^−1^	I_A_, %	I_B_,%	τ_avg_, ps
0.10	1.16	147	240	106	19.1 ± 0.4	0.04 ± 0.08	86.7	0.2	149
0.15	1.09				15.4 ± 0.1	0.21 ± 0.03	83.4	1.0	146
0.20	1.12				21.0 ± 0.2	0.96 ± 0.04	84.0	3.5	159
0.25	1.17				20.2 ± 0.5	0.61 ± 0.05	84.9	2.3	155

## Data Availability

The data presented in this study are available on request from the corresponding author. The data are not publicly available due to privacy reasons.

## Supplementary Materials

The following supporting information can be downloaded at: https://www.mdpi.com/article/10.3390/ma15031017/s1, Figure S1: PALS spectra for GCr15 steel after high-speed cutting, Figure S2: DBS spectra for GCr15 steel after high-speed cutting, Figure S3: CDBS spectra for GCr15 steel after high-speed cutting.
