# Supplementary material for: Positron Annihilation Spectroscopy Study of Metallic Materials after High-Speed Cutting
Source: Materials (Basel). 2022 Jan 28;15(3):1017. doi: 10.3390/ma15031017 (PMC8838609; doi:10.3390/ma15031017)
Supplement: Supplementary file 1 [file materials-15-01017-s001.zip › materials-1533038-supplementary.pdf]

Positron Annihilation Spectroscopy Study of Metallic Materials after High-Speed Cutting

Jinquan Li [1,*], Roman Laptev [2,*], Iurii Bordulev [2], Krzysztof Siemek [3,4], Pawel Horodek [3,4], Shen Haolun [1], Anton Lomygin [2] and Jian Cui [2]

[1] School of Mechanical Engineering, Shenyang Ligong University, No.6 Nanping Center Road,
Hunnan New District, Shenyang 110159, China; shenhaolun0127@163.com (S.H.)
[2] Division for Experimental Physics, National Research Tomsk Polytechnic University, 634050 Tomsk, Russia; bus@tpu.ru (I.B.); lomyginanton141@gmail.com (A.L.); ttszyan@mail.ru (J.C.)
[3] Institute of Nuclear Physics Polish Academy of Sciences, PL-31342 Krakow, Poland; siemek.krzysztof@gmail.com (K.S.); pawel.horodek@ifj.edu.pl (P.H.)
[4] Joint Institute for Nuclear Research, Joliot-Curie 6, 141980 Dubna, Moscow Region, Russia
* Correspondence: li_jinquan@163.com (J.L.); laptevrs@tpu.ru (R.L.);
Tel.: +7-913-852-3733 (R.L.)

Supplementary materials

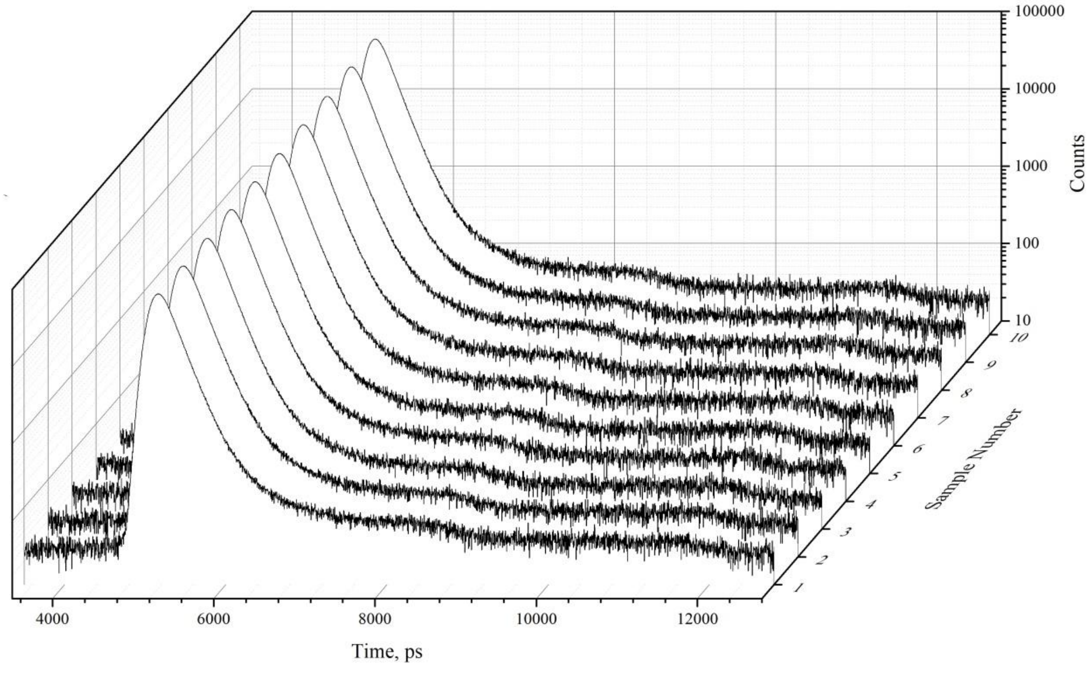

Figure S1. PALS spectra for GCr15 steel after high-speed cutting.

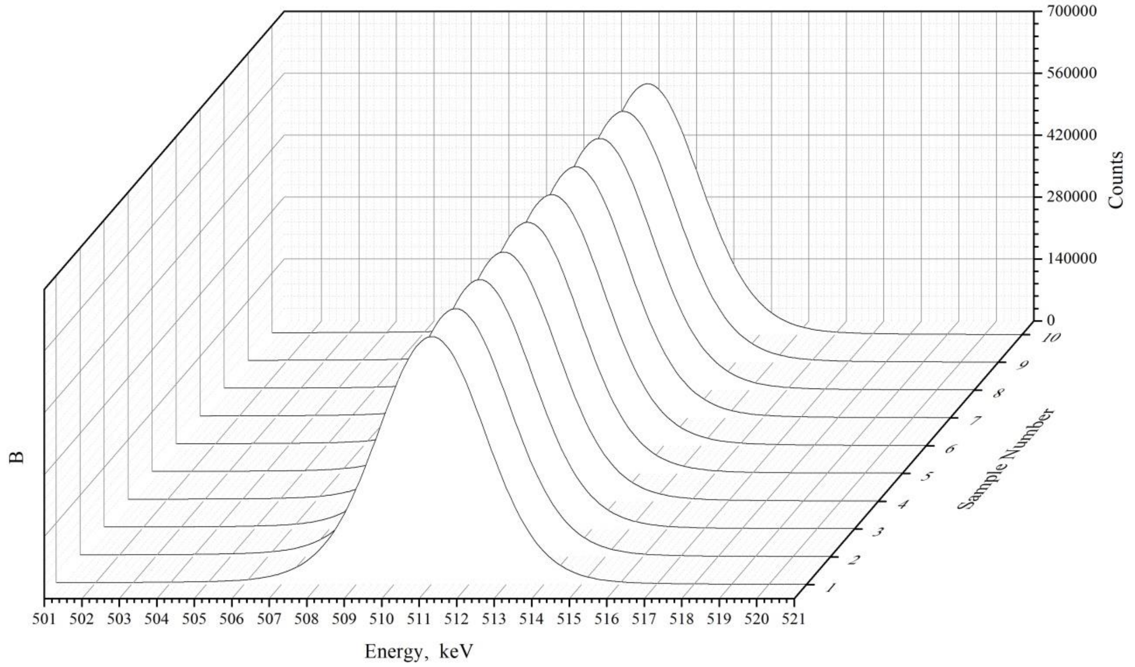

Figure S2. DBS spectra for GCr15 steel after high-speed cutting.

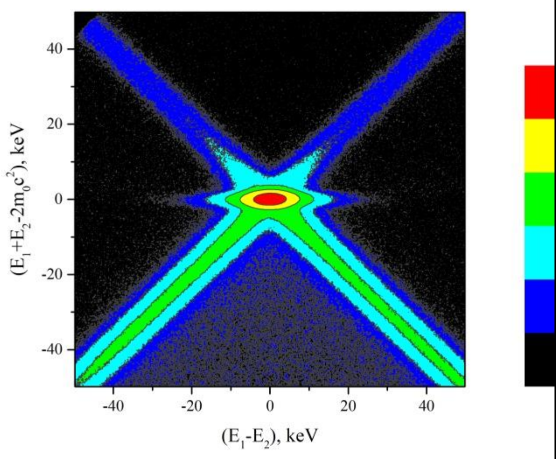40<br>20<br>0<br>-20<br>-40<br>(E1+E2-2m0c2), keV<br>(E1-E2), keV	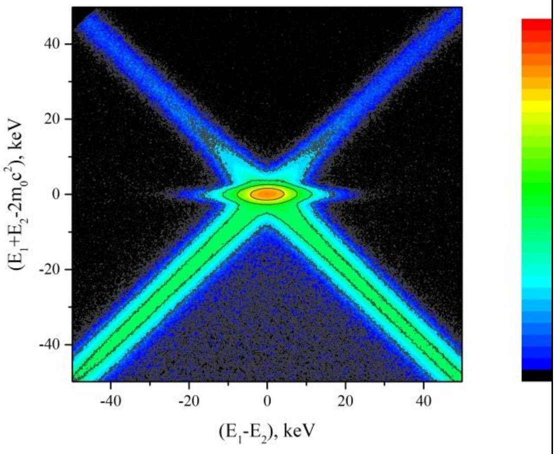40<br>20<br>0<br>-20<br>-40<br>(E1+E2-2m0c2), keV<br>(E1-E2), keV
**#1**	**#2**
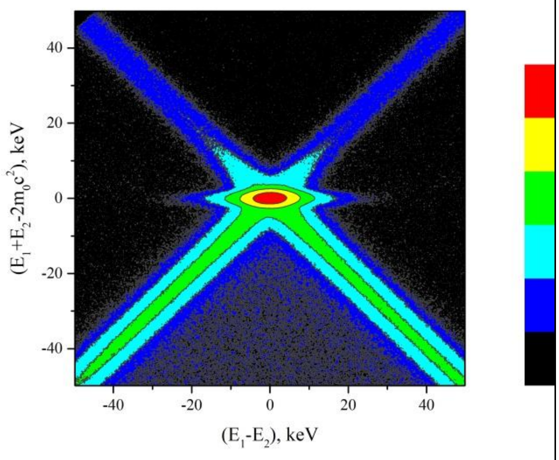40<br>20<br>0<br>-20<br>-40<br>(E1+E2-2m0c2), keV<br>(E1-E2), keV	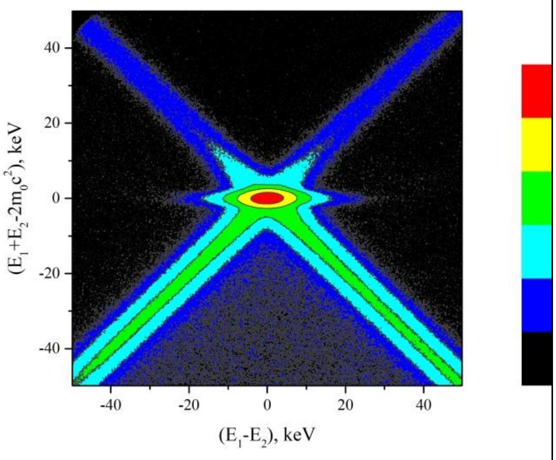40<br>20<br>0<br>-20<br>-40<br>(E1+E2-2m0c2), keV<br>(E1-E2), keV
**#3**	**#4**
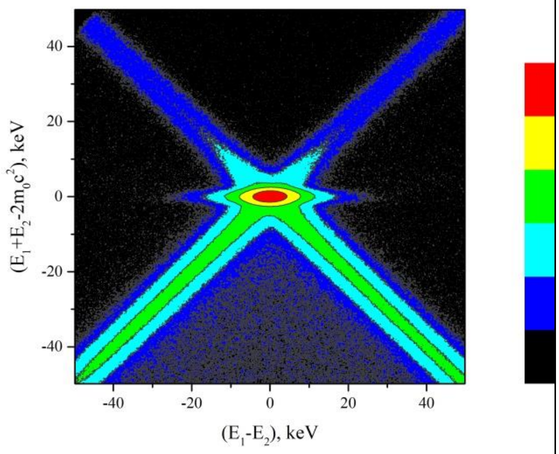40<br>20<br>0<br>-20<br>-40<br>(E1+E2-2m0c2), keV<br>(E1-E2), keV	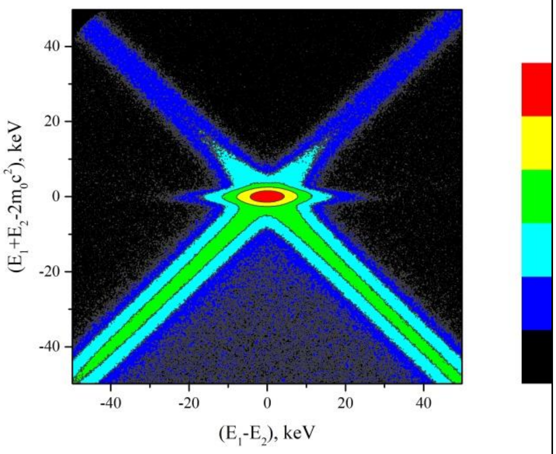40<br>20<br>0<br>-20<br>-40<br>(E1+E2-2m0c2), keV<br>(E1-E2), keV

#5	#6

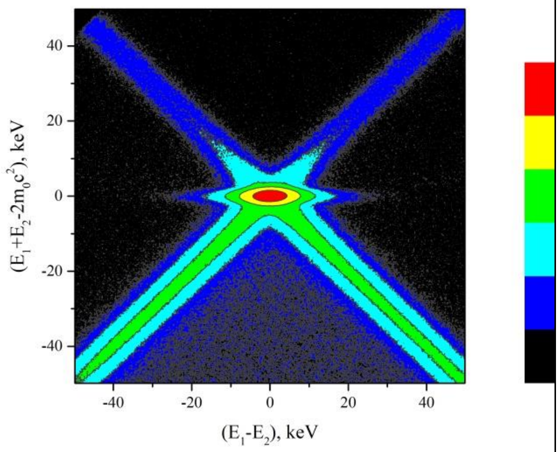

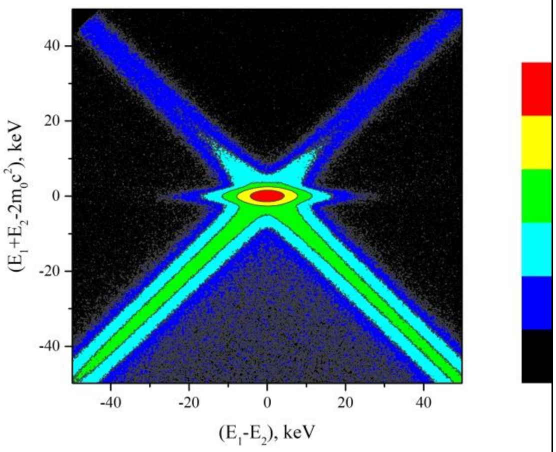

#7	#8

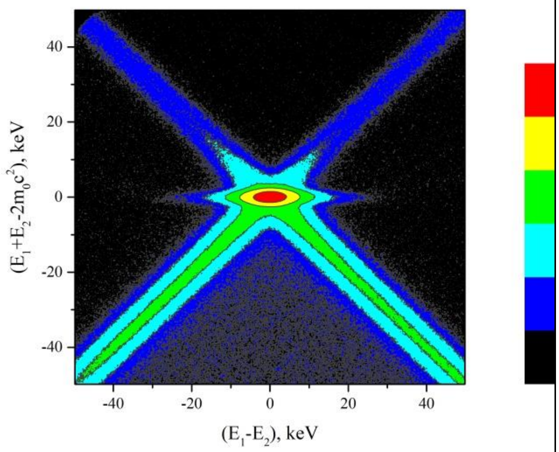

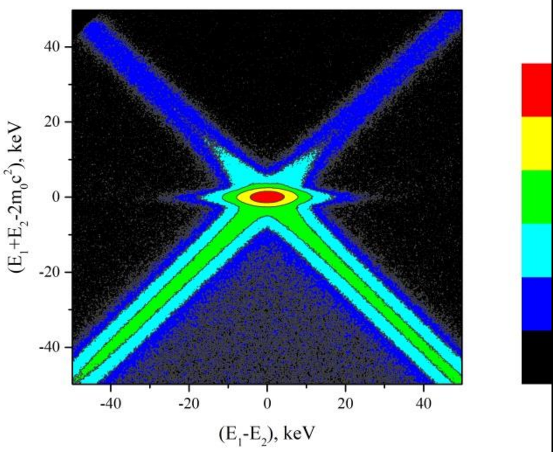

#9	#10

Figure S3. CDBS spectra for GCr15 steel after high-speed cutting.
